# Creating flexible motor memories in human walking

**DOI:** 10.1038/s41598-017-18538-w

**Published:** 2018-01-08

**Authors:** Kristan A. Leech, Ryan T. Roemmich, Amy J. Bastian

**Affiliations:** 1 Department of Neuroscience, The Johns Hopkins University School of Medicine, Baltimore, MD 21205 USA; 2 Center for Movement Studies, Kennedy Krieger Institute, Baltimore, MD 21205 USA; 3 Department of Physical Medicine and Rehabilitation, The Johns Hopkins University School of Medicine, Baltimore, MD 21205 USA

## Abstract

The human nervous system has the ability to save newly learned movements (i.e. re-learn faster after initial learning) and generalize learning to new conditions. In the context of walking, we rely on savings and generalization of newly learned walking patterns to navigate changing environments and make progressive improvements with gait rehabilitation. Here, we used a split-belt treadmill to study how different perturbation parameters can influence savings and generalization of learning during walking. In Experiment 1, we investigated the effect of split perturbation size on savings of a newly learned walking pattern. We found that larger perturbations led to better savings than smaller perturbations. In Experiment 2, we studied how different features of the initial split perturbation influenced the generalization of learning. Interestingly, we found that practicing the same thing twice did not lead to fastest learning. Instead, initial exposure to larger perturbation ratios led to faster subsequent learning of smaller perturbation ratios as compared to repeated exposures to small perturbations. Collectively, our findings reveal that initial learning conditions can be leveraged to increase savings and shape flexible motor memories during walking.

## Introduction

In human motor learning, we often emphasize savings and generalization as learning phenomena with important clinical relevance. For example, savings – i.e., the ability to store and quickly recall learned movements – drives successful rehabilitation because therapy hinges on the ability to build upon what has been learned previously. And if rehabilitation is to be progressive, people must generalize learning to new contexts outside of the specific training environment. This leads to an important question: what is the “best” way to learn if we hope to maximize savings and generalization?

Many studies have shown that movements learned via motor adaptation, or error-based learning, can be quickly recalled even after the new pattern is unlearned and performance has returned to baseline^1–3^. This savings manifests behaviorally as accelerated re-learning when the same task is practiced twice. Importantly, studies of savings have demonstrated that the way we learn new movements affects how quickly we re-learn them in the future. Our previous work has shown that savings during walking can be driven by (1) previous exposure to an abrupt versus gradual change in walking conditions (i.e. perturbations) or (2) prolonged practice in the new walking condition^4^. Data collected in reaching adaptation tasks has similarly demonstrated that the perturbation schedule can influence savings of a newly learned movement^5^ and further suggests that large perturbations during initial learning are also necessary to induce savings^6^.

Motor adaptation also generalizes to novel conditions. That is, learning does not necessarily start anew each time an individual encounters a new environment. Studies of adapted arm or hand movements have demonstrated that prior learning of similar tasks can also accelerate learning of untrained movements or within new task demands^7–9^. Similar to savings of a learned movement, generalization of learning is also influenced by how we initially learn^10^, with some evidence for greater generalization following learning in response to larger perturbations^11,12^.

Here we investigated parameters of learning during walking that facilitate (1) greater savings of a newly learned walking pattern and (2) better generalization to new task demands. First, we systematically varied the learning environment by manipulating the size of the perturbation driving adaptation. From this we demonstrated that savings of a new walking pattern is augmented through exposure to larger perturbations. We then show that prior learning of a novel walking pattern in response to large perturbations not only generalized to new task demands, but led to even faster ‘re-learning’ than could be achieved by practicing the same thing twice. In sum, our findings demonstrate that repeated practice is not necessarily the best way to learn a new movement pattern, but rather specific learning parameters can be leveraged to accelerate subsequent learning in familiar or novel conditions.

## Results

### Experiment 1: How does perturbation size affect savings of a newly learned walking pattern

**Figure 1:**
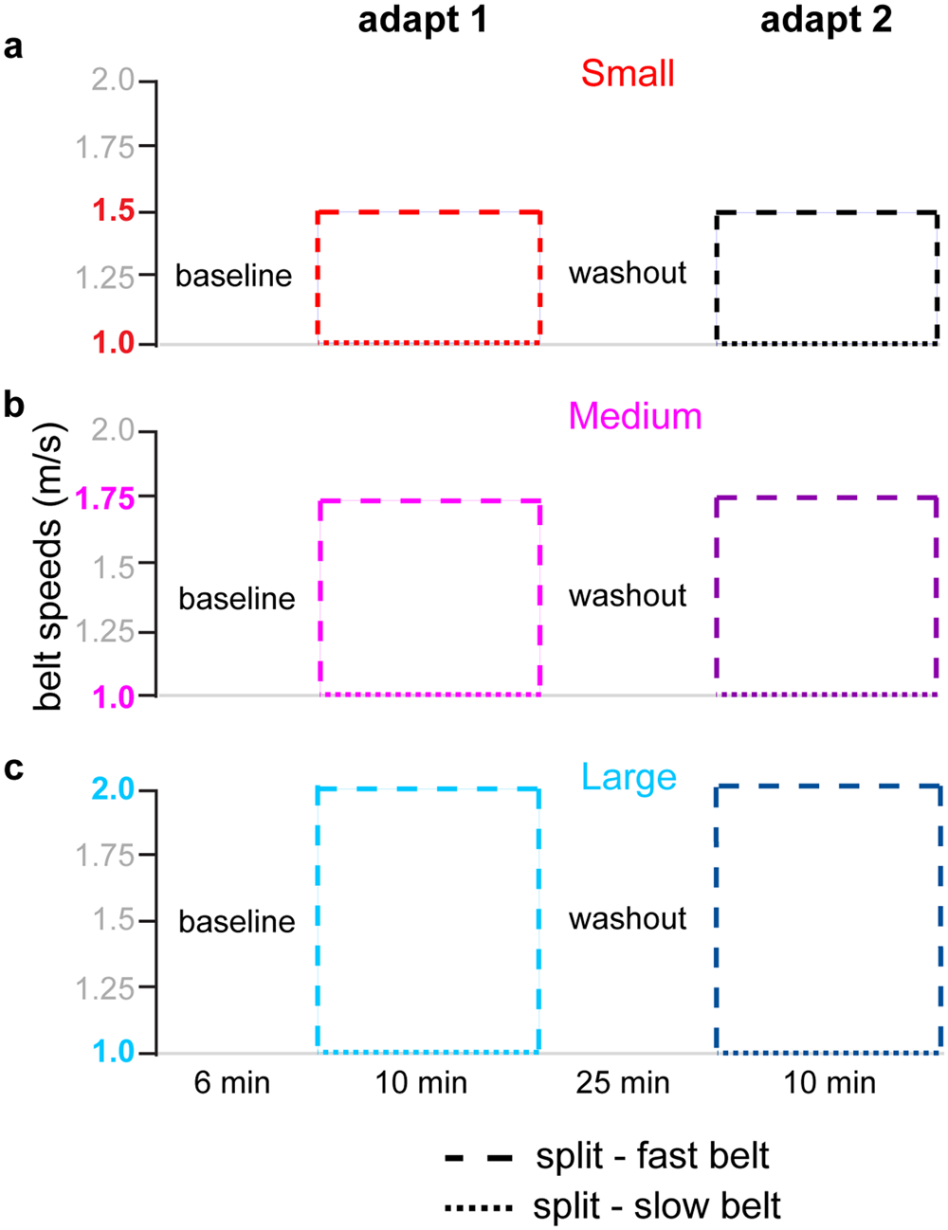
Experiment 1 protocol diagrams are shown for the Small (**a**), Medium (**b**), and Large (**c**) groups. Dashed lines indicate the speeds of the fast and slow belts, with solid lines indicating when the belts are tied. Colors assigned to each group and adaptation block will be constant throughout the figures.

The goal of Experiment 1 was to understand how perturbation size affects savings of a newly learned walking pattern. In other words, we wondered whether people would re-learn a walking pattern faster upon a second exposure if they experienced larger perturbations during initial learning. We studied three groups of healthy young adults while they walked on a split-belt treadmill. All groups performed a conventional savings paradigm consisting of tied-belt baselines, an initial split-belt learning bout (adapt 1), tied-belt washouts, and then a second split-belt learning bout (adapt 2; Fig. 1). To study the effect of the size of the perturbation on savings, we varied the split-belt ratio among groups. In the Small group (n = 16) the split-belt ratio was 1.5:1. The Medium group (n = 16) walked with the belts moving at a 1.75:1 ratio. Finally, the Large group (n = 16) experienced a 2:1 split ratio (see Fig. 1 for further details on split-belt configurations). All groups underwent tied-belt baseline and washout periods at the slow and fast speeds that they experienced during the split-belt learning bouts. Given reports of persistent motor memories^13^, to ensure complete unlearning during washout we specifically designed the experiments here to have an extended washout relative to other studies of savings during walking (i.e. 25 versus 10 minutes^2,4^). Of note, the washout blocks also alternated between fast and slow speeds because previous work has shown that motor aftereffects can persist at both speeds^14^.

We assessed learning and re-learning by measuring step length asymmetry (SLA) because this gait parameter adapts robustly to split-belt walking perturbations^15^ and shows savings across multiple learning bouts^2,4^. SLA is calculated using the following equation:

SLA = (fast step length−slow step length)/(fast step length + slow step length)

SLA value of 0 indicates symmetric stepping. We confirmed that all groups walked with similar SLA during baseline (F_(2,45)_ = 1.09, p = 0.36).

**Figure 2:**
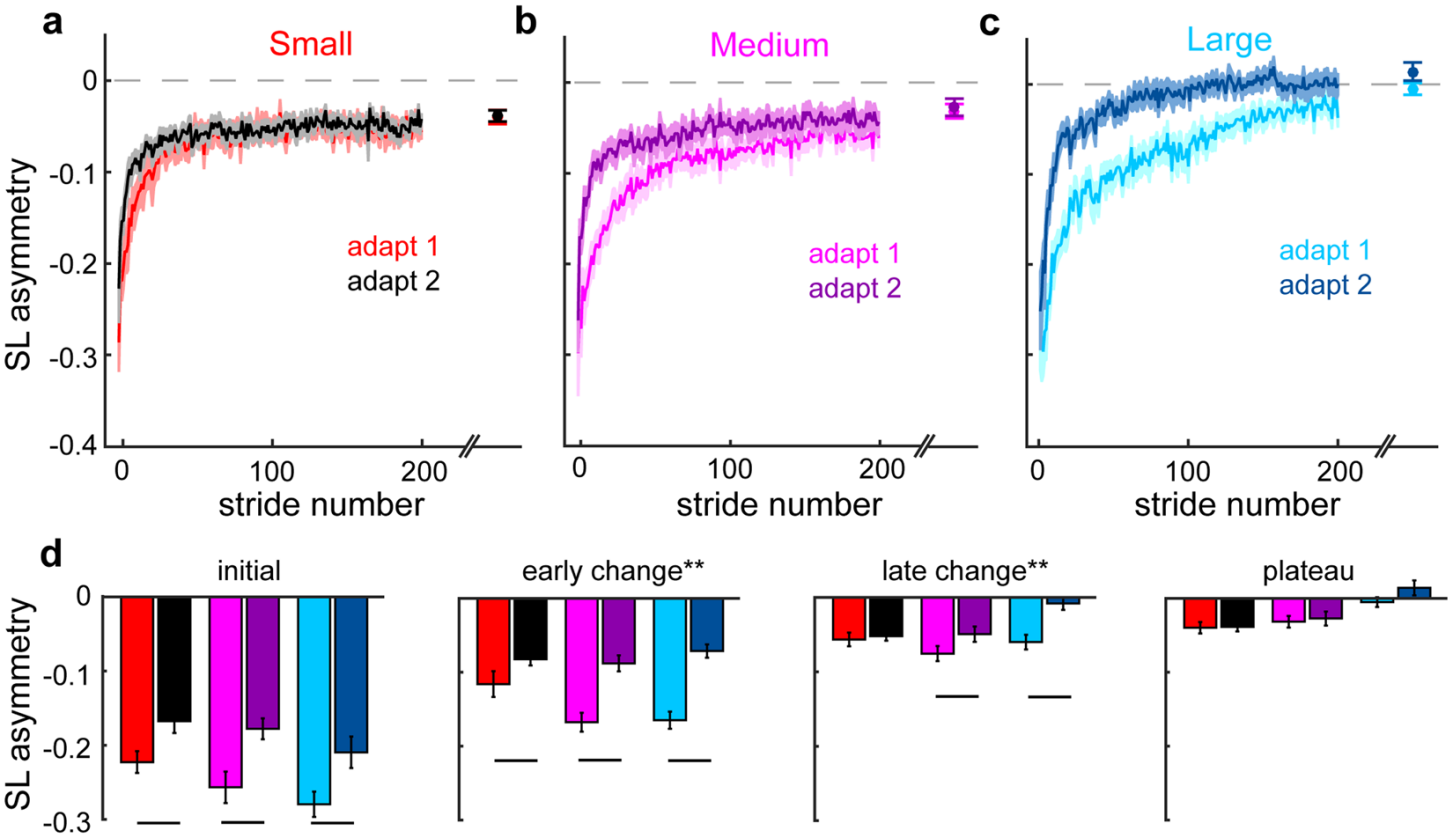
Comparison of step length asymmetry (SLA) during adapt 1 and adapt 2 (i.e. savings) among the Small (red and black traces) (**a**), Medium (pink and purple traces) (**b**), and Large (cyan and blue) (**c**) perturbation groups in Experiment 1. Mean curves across participants within each group ± SE are shown. The curves are truncated in length to match the participant that took the fewest strides during each condition. Data points immediately following the adapt 1 and adapt 2 curves show the SLA during the plateau (mean ± SE of the last 30 strides) for each group. (**d**) Bar graphs are shown indicating the SLA during initial (mean of the first 5 strides), early change (mean of strides 6–30), late change (mean of strides 30–200), and plateau of adapt 1 and adapt 2 for each group (mean ± SE). **Indicates a significant group*learning bout interaction effect for that epoch (P < 0.05). Lines indicate significant differences between adapt 1 and adapt 2 found with LSD posthoc tests (P < 0.05). The statistical analyses are described in Methods.

We found that perturbation size affects the magnitude of savings. Figure 2a–c shows the group mean timecourses for SLA during the adapt 1 and adapt 2 learning bouts with the Small group exhibiting the least savings and the Large group showing the most. To evaluate the difference in savings we compared specific epochs within these curves (Fig. 2d).

We found similar decreases in initial asymmetry from adapt 1 to adapt 2 across all three groups (main effect of learning bout, F_(1,45)_ = 37.4, P < 0.0001, main effect of group, F_(2,45)_ = 2.81, P = 0.07, group*bout interaction, F_(2,45)_ = 0.39, P = 0.68). However, the differences in savings started to emerge in our measure of early change in SLA. Specifically, we saw a group*bout interaction (F_(2,45)_ = 6.53, P = 0.003) such that the Medium and Large groups showed more change from adapt 1 to adapt 2 compared to the Small group. That said, all groups showed significant decreases in SLA during early change in adapt 2 as compared to adapt 1 (LSD post hoc: Small P = 0.009, Medium P < 0.0001, and Large P < 0.0001). The group*bout interaction was most evident in SLA during late change, with only the Medium and Large perturbation groups demonstrating significant decreases in SLA from adapt 1 to adapt 2 (group*bout interaction, F_(2,45)_ = 8.94, P = 0.001; LSD post hoc comparing late change during adapt 1 to that during adapt 2: Small P = 0.582, Medium P = 0.002, and Large P < 0.0001).

**Figure 3:**
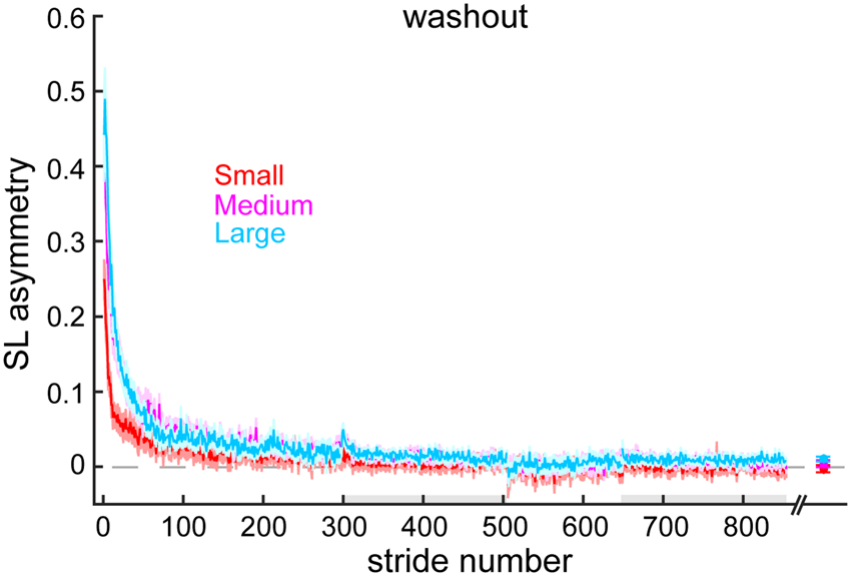
Changes in step length asymmetry during washout blocks following adapt 1. Grey shading along the x-axis indicates the SLA during washout at fast vs slow speeds. Mean curves across participants within each group ± SE are shown. Note that the curves are truncated in length to match the participant that took the fewest strides during each washout block. Data points immediately following the last washout block represent the SLA at the end of washout (mean ± SE of the last 30 strides) for each group. There were no significant differences in SLA among the groups at the end of washout.

We also considered that the differences in savings among groups might be explained by (1) differences in the degree of symmetry attained by the end of adapt 1 (i.e., some groups may have learned “more”) or (2) differences in the amount of unlearning during washout (i.e., some groups may not have fully unlearned). First, because we observed that the Large group stepped more symmetrically than the Small and Medium groups by the end of adapt 1 (adapt 1 plateau: F_(2,45)_ = 5.98, P = 0.005; Bonferroni post hoc: Small vs. Medium: P = 1.0, Small vs. Large: P = 0.006, Medium vs. Large: P = 0.045), we performed a one-way ANCOVA to compare the amount of savings (specifically, the difference in late change from adapt 1 to adapt 2) among the groups while controlling for the adapt 1 plateau. When we included adapt 1 plateau as covariate, we still observed a significant difference in savings between the groups, with post hoc tests demonstrating significant differences between the Large and Small, but not Medium perturbation groups (F_(2,44)_ = 6.56, P = 0.003; Bonferroni post hoc: Small vs. Medium: P = 0.21, Small vs. Large: P = 0.002, Medium vs. Large: 0.15). Therefore, the perturbation size provided is the primary predictor of savings with little influence of the amount of symmetry restored during adapt 1. Second, we compared the SLA during baseline and last washout blocks and found that all groups were similarly washed out (no group*time interaction effect: F_(2,45)_ = 0.40, P = 0.67) prior to adapt 2 (Fig. 3), and thus the degree of washout is unlikely to have affected the differences in savings we observed among groups.

In summary, the results from Experiment 1 revealed that larger perturbations lead to better savings than smaller perturbations.

### Experiment 2: Is practicing the same thing twice always the fastest way to learn a new walking pattern

**Figure 4:**
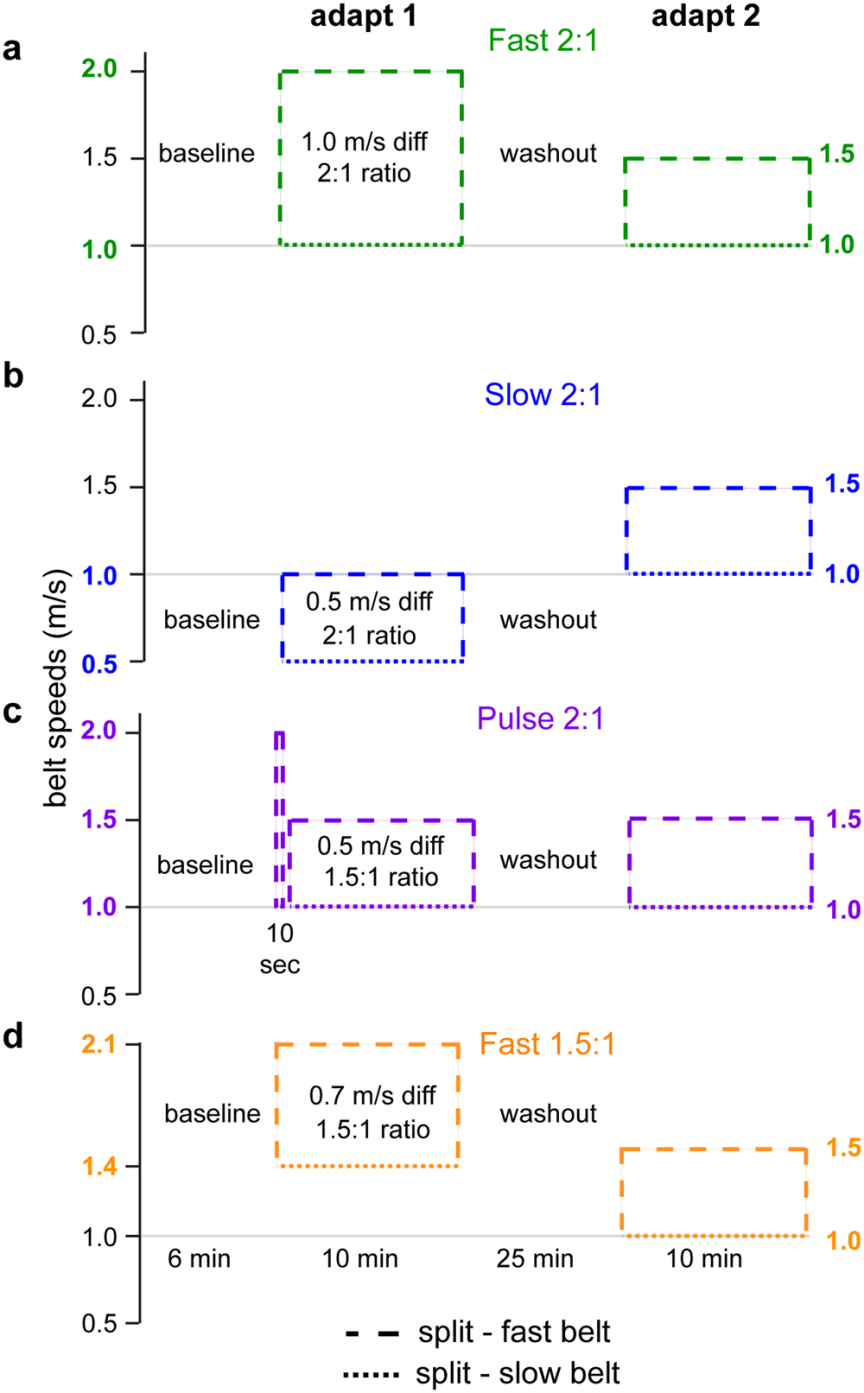
Experiment 2 protocol diagrams are shown for the Fast 2:1 (**a**), Slow 2:1 (**b**), Pulse 1.5:1 (**c**), and Fast 1.5:1 (**d**) groups. Dashed lines indicate the speeds of the fast and slow belts, with solid lines indicating when the belts are tied. Embedded in the adapt 1 learning bout schematic is text highlighting the adapt 1 split parameters manipulated for that group. Note that the adapt 2 bout is kept consistent across groups. Colors assigned to each group will be constant throughout the figures.

Intuitively, one might think that the fastest way to learn a new walking pattern is by practicing the same pattern repeatedly. However, our findings from Experiment 1 question whether this is actually true. We tested this by studying four groups of healthy young adults (n = 16/group) and manipulating different features of the split-belt perturbations in adapt 1 to see which features influenced learning during adapt 2. Specifically, we manipulated the belt speed ratio, average belt speed, and belt speed difference such that each group experienced a “large perturbation” through at least one of these parameters in adapt 1(i.e., large ratio, fast average speed, or large speed difference). Then, the adapt 2 condition was consistent across groups and identical to the Small group from Experiment 1 (i.e. a 1.5:1.0 m/s split, see Fig. 4 for details). Recall that the Small group did not show as much savings in Experiment 1.

The groups we studied are as follows (Fig. 4). The Fast 2:1 group walked with a large belt speed ratio, an intermediate average belt speed, and a large belt speed difference (2.0 m/s:1.0 m/s). The Slow 2:1 group walked with a large belt speed ratio, a slow average belt speed, and a small belt speed difference (1.0 m/s:0.5 m/s). Thus we held the ratio constant in these two groups, but varied the average belt speed and belt speed difference. The Pulse 1.5:1 group received a 10 second exposure to a large perturbation (2.0 m/s:1.0 m/s) and then returned to walking at a smaller perturbation (1.5 m/s:1.0 m/s) for the remained of adapt 1. This was designed to specifically test role of initial asymmetry size. The Fast 1.5:1 group walked with a small belt speed ratio, a fast average belt speed, and an intermediate belt speed difference (2.1 m/s:1.4 m/s). This allowed us to assess the influence of faster speeds in absence of a large ratio.

Since each group had an “advantage” in at least one parameter (i.e., large ratio, fast average speed, or large speed difference), we were able to investigate the effect of each parameter on the generalization of learning to novel walking conditions in adapt 2. We did this by comparing the adapt 2 performance in these groups to the adapt 2 data of the Small group in Experiment 1. In other words, we wondered if differences in adapt 1 would accelerate the learning during the same adapt 2 that showed the least savings in Experiment 1.

**Figure 5:**
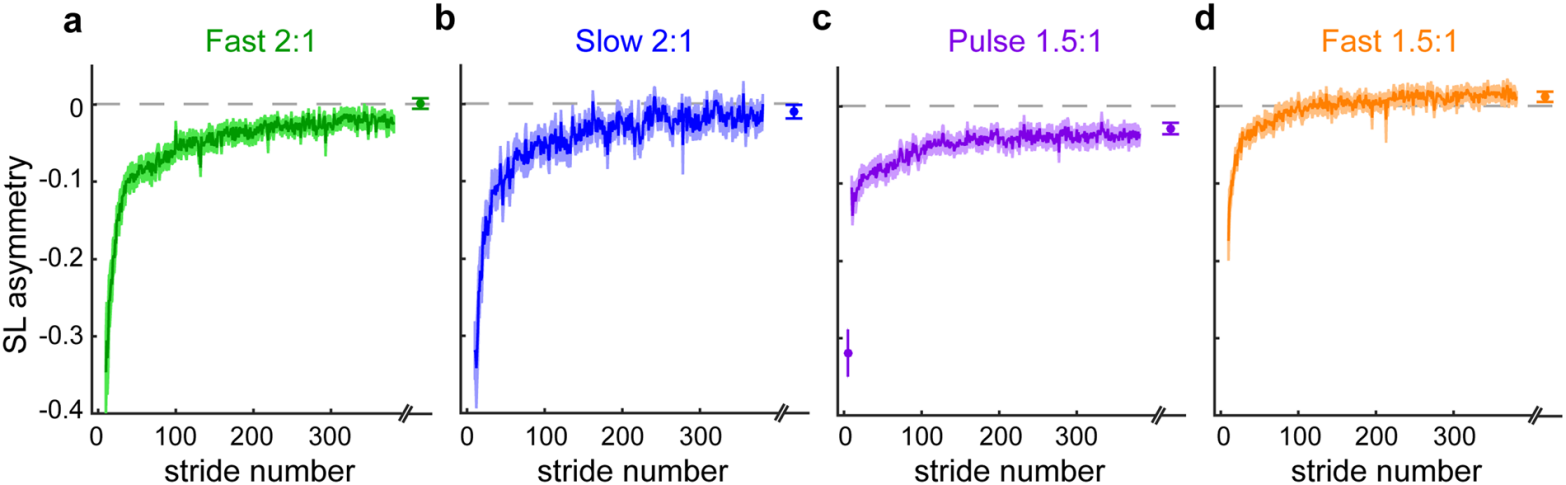
Changes in step length asymmetry during adapt 1. Mean curves across participants within each group ± SE are shown. The curves are truncated in length to match the participant that took the fewest strides during each condition. Data points immediately following the curves show the SLA during the plateau (mean ± SE of the last 30 strides) for each group. Stepping behaviors in each group are different, reflecting the varied learning parameters in this initial learning block. Note that the Fast 2:1 (**a**), Slow 2:1 (**b**), and Pulse 1.5:1 (**c**) groups exhibit similar magnitudes of asymmetry initially, while the Fast 1.5:1 group (**d**) shows a smaller initial asymmetry. Additionally, each group plateaued to different levels of asymmetry by the end of adapt 1.

We did not observe any group differences in SLA among groups during baseline walking (F_(4,75)_ = 1.54, P = 0.2). We show SLA during the unique adapt 1 conditions for each group in Fig. 5. The Fast 2:1 and Slow 2:1 groups showed large asymmetries early (Fig. 5a,b), as did the initial pulse within the Pulse 2:1 group (Fig. 5c). The Fast 1.5:1 group showed less asymmetry early on and appear to plateau at a more symmetric level (Fig. 5d).

**Figure 6:**
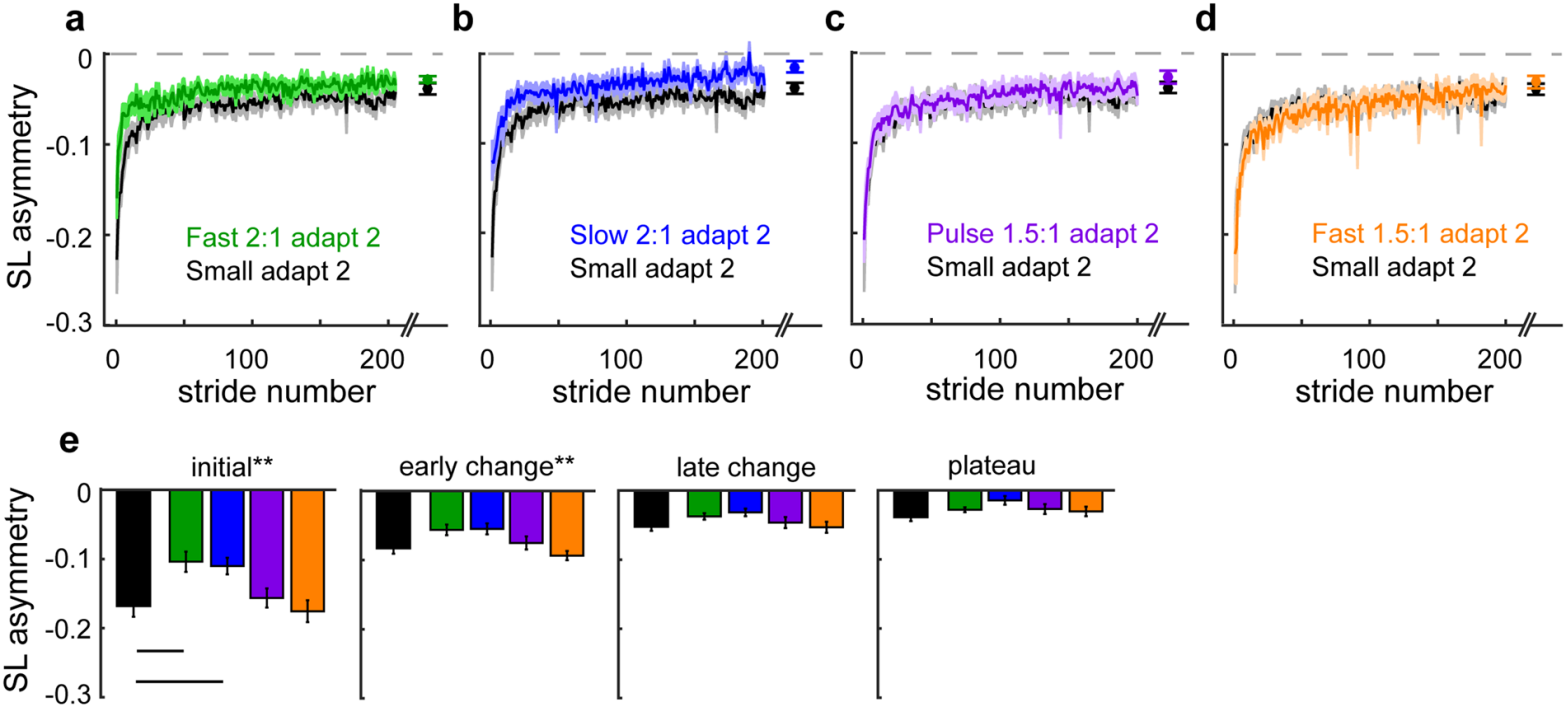
Comparison of step length asymmetry during adapt 2 relative to the adapt 2 behavior of the Small perturbation group in Experiment 1 (black). Plots are organized such that groups that received a 2:1 split ratio and exhibit accelerated learning relative to the Small group are on the left (**a** and **b**) and group exposed to 1.5:1 ratio and demonstrate the same behavior as the Small group in adapt 2 are on the right (**c** and **d**). Mean curves across participants within each group ± SE are shown. The curves are truncated in length to match the participant that took the fewest strides during each condition. Data points immediately following the curves show the SLA during the plateau (mean ± SE of the last 30 strides) for each group. Differences among the groups during initial, early change, late change, and plateau are quantified in the bar plots at the bottom (E; mean ± SE). **Indicates a significant one-way ANOVA for that epoch (P < 0.05). Lines indicate a significant difference in SLA relative to the Small perturbation group from Experiment 1 that was found with Dunnett’s post hoc test (P < 0.05). The statistical analyses are described in Methods.

Figure 6 shows that the large ratio split-perturbation led to accelerated ‘re-learning’ of a novel walking pattern (‘re-learning’ in quotations because only the Small group from Experiment 1 had actually experienced the perturbation applied during adapt 2). Specifically, the Fast 2:1 and Slow 2:1 groups both demonstrated faster initial and early changes in SLA during adapt 2 than the Small group in Experiment 1, despite the fact that the Small group experienced the exact same perturbation twice (Fig. 6e; initial: F_(4,75)_ = 5.28, P = 0.001; Dunnett’s post hoc: Fast 2:1 P = 0.01, Slow 2:1 P = 0.02; early change: F_(4,75)_ = 4.23, P = 0.004; Dunnett’s post hoc: Fast 2:1 P = 0.08, Slow 2:1 P = 0.06; late change: F_(4,75)_ = 2.0, P = 0.104). This suggests that the beneficial effect of a large perturbation is mediated by the size of the split ratio, as the Fast 2:1 and Slow 2:1 groups were matched for ratio while the belt speed difference and average belt speed varied. The importance of the split ratio size was further confirmed by our finding that the other two groups showed no added benefit of faster average speed (Dunnett’s post hoc: Fast 1.5:1, initial: P = 0.99, early change: P = 0.74) or large initial asymmetry alone (Dunnett’s post hoc: Pulse 1.5:1, initial: P = 0.95, early change: P = 0.92) when these conditions were paired with extended learning at a small split ratio. These results suggest that initial learning in response to larger split ratios not only generalizes to novel conditions, but also can lead to even faster learning than practicing the same thing twice.

**Figure 7:**
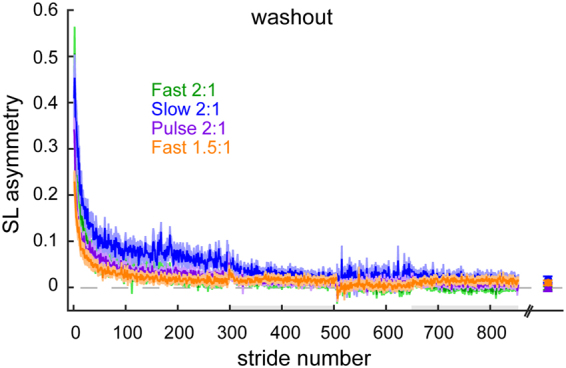
Mean curves of step length asymmetry during washout blocks after adapt 1 for each group (mean ± SE). Note that the curves are truncated in length to match the participant that took the fewest strides during each washout block. Data points immediately following the last washout block represent the SLA at the end of washout (mean ± SE of the last 30 strides) for each group. There were no significant differences in SLA among the groups at the end of washout. Grey shading along the x-axis indicates the strides taken during washout at fast washout blocks.

Similar to Experiment 1, we checked to see if these effects could be explained by any differences in the level of washout between groups. Changes in SLA during Experiment 2 washout are presented in Fig. 7. We found small differences in SLA across the groups (main effect for group: F_(4,75)_ = 3.09, P = 0.02) as well as differences between baseline and the end of washout (main effect for time: F_(1,75)_ = 4.79,P = 0.032), but these differences were consistent across groups (no group*time interaction effect: F_(4,75)_ = 0.68, P = 0.61). Therefore, unequal washout between groups does not explain our results.

In summary, the results from Experiment 2 revealed that first practicing something different, and perhaps “harder”, can actually facilitate faster learning of a specific movement as compared to repetitive practice of the same task demands.

## Discussion

The human nervous system has the remarkable ability to learn, store, and recall new movement patterns. These features not only enable us to successfully navigate and interact with our surroundings, but are also critical to the success of rehabilitation targeted to improve motor function. Here, we present new information about the features of initial learning that drive savings and generalization of learning during walking. First, we found that the size of the perturbation provided affected the degree to which a new walking pattern is saved. Though all subjects had some savings of the newly learned walking pattern, subjects exposed to a large perturbation demonstrated the greatest savings upon re-exposure. Next, we demonstrated that practicing the same thing twice was not the best way to speed learning of a new walking pattern. Instead, larger perturbation ratios on the first exposure sped the learning of smaller perturbation ratios on the second exposure. Our findings reveal that locomotor learning produces flexible motor memories that are shaped largely by initial learning conditions.

We first showed that participants exhibited greater savings with larger perturbations during walking. Savings of a new motor behavior following error-based sensorimotor learning has been largely viewed to be driven by a cerebellar-dependent mechanism^16^. As such, multiple error-based learning models have been proposed to explain the phenomenon of savings in arm and hand movements (e.g. refs^17,18^), but none of these fully account for our results of greater savings with larger perturbations. Recent evidence suggests that cognitive learning processes interact with error-based learning to account for similar findings in reaching. For example, savings during reaching movements only occurs with large perturbations and this gain in performance was facilitated by use of a cognitive aiming strategy upon re-exposure to a large perturbation^6^. This is consistent with data that demonstrate an increased contribution of explicit learning processes as task demands or perturbation sizes increase during sensorimotor learning^19^. Furthermore, we have previously shown that savings during walking that occurs following large abrupt perturbations may be driven acquisition of a more accurate explicit knowledge of the learning environment^4^.

Though studies of reaching suggest that aiming strategies may be important for savings, we think it is unlikely that this accounts for the effects that we see in walking. When walking over level surfaces, locomotion is a relatively automatic, patterned behavior that does not require attention to where we “aim” our feet. Rather, attention to precise foot placement only increases when environmental demands, such as obstacles, are present^20–22^. These observations suggest that explicit aiming strategies contribute less to savings during waking as compared that reported with reaching movements. However, we cannot entirely rule this out because there is no clear method to measure the contribution of an explicit aiming strategy versus the more automatic learning process that occurs during this walking task. An attempt to do so would change the task from an automatic walking task to a precision stepping task. While we are skeptical of a role for aiming strategies, our recent work demonstrates that other explicit components of learning contribute to savings during walking. Specifically, we found that savings relies on being able to explicitly recall the perturbation^4^, even if subjects cannot verbalize how they alter their walking.

Results from Experiment 2 demonstrated that prior exposure to a similar task led to generalization of learning to walking within new conditions. All groups performed at least as well as the reference behavior (the group that practiced the same thing twice) regardless of initial learning parameters. This is consistent with the well-documented ability of the motor system to undergo “meta-learning” and generalize prior learning to improve performance within novel task conditions (e.g. refs^9,23,24^). Multiple mechanisms have been proposed to account for this phenomenon. For example, Flanagan and colleagues suggested that the generalization of prior learning to novel task conditions is due to the ability of the central nervous system to alter previously acquired internal models to meet new requirements of a similar task^8^. Additionally, a model for motor learning has recently been proposed that attributes the meta-learning phenomenon to an increased sensitivity to and memory of previously experienced errors^18^. There is also data to suggest that subjects learn the structural features of a task, which speeds subsequent learning of novel tasks that have a shared structure^25^. While this work offers evidence for candidate mechanisms that may have contributed to the generalization of learning demonstrated here, it was performed in the context of upper extremity movements. As such, further work is required to determine the degree to which these or any other mechanisms also contribute to the generalization of learning during walking.

Beyond a global generalization of learning to new task conditions, we found that practicing the same thing twice is not necessarily the fastest way to learn a new walking pattern. Specifically, initial exposure to large ratio perturbations drove faster ‘re-learning’ than could be achieved by repeat exposure to small perturbations. We find two details about this result thought provoking. First, this suggests that initially practicing something more perturbing or difficult may lead to better performance than repeated practice of a specific task. Much of the initial work exploring the generalization of learning in adapted arm or hand movements suggests that prior exposure to a similar perturbation, regardless of size, facilitates better performance with subsequent adaptation^9,12^. Yet, similar to our results, recent work suggests that increasing the difficulty of initial learning can facilitate better generalization of learning to different task demands. For example, learning following practice of reaching movements to variable versus fixed targets better generalizes to untrained targets^10^. Additionally, high versus low acceleration slip training on a treadmill has been shown result in greater control in response to untrained slip directions^26^ and overground slips^11^. Yet, this effect may be restricted to certain forms of added task difficulty, as divided attention during initial learning has been shown to limit generalization of learning^27^.

One puzzling detail remains: why is the ratio of the belt speeds the perturbation parameter that improves the generalization of learning to new conditions while the absolute speeds appear to have little influence? Given that walking requires interlimb coordination characterized by an out-of-phase relationship between the limbs, a relative versus absolute speed difference between the belts may be a more salient perturbation parameter. However, further work is necessary to determine the mechanism that underlies this result. Interestingly, this indicates that savings and generalization can be driven by high ratio perturbations at slower speeds, which may prove to be particularly important within clinical populations that may not tolerate walking at faster speeds.

In summary, here we determined that the size of the perturbation driving the learning influences the degree to which a new walking pattern is saved. We also found that learning of a specific walking pattern was fastest, not by repeated exposure to the same task, but through the generalization of prior learning in response to larger perturbations. These results help clarify the structure of practice that can facilitate savings and generalization learning during walking. As such, these findings may be relevant when considering the design of rehabilitation interventions that leverage adaptive motor learning mechanisms to address gait deficits. Future work will be required to translate these results into patient populations and determine the clinical relevance.

## Methods

### Participants

One hundred and twelve young healthy volunteers participated in this study (Experiment 1: n = 48, 17 males, 31 females; mean age ± SD: 23 ± 5 yr; Experiment 2: n = 64, 17 males, 47 females; mean age ± SD: 23 ± 4 yr). All participants were naïve to split-belt treadmill walking, participated in only one experiment, and provided informed written consent before participating. The protocols were approved by the Johns Hopkins Medicine Institutional Review Board and all experiments were performed in accordance with the relevant guidelines and regulations.

### Experimental procedures

During both experiments, participants walked on a custom-built split-belt treadmill (Woodway USA, Waukesha, WI) that has two separate belts driven by independently controlled motors. The treadmill motors were controlled using a custom-written MATLAB code (The Mathworks, Natick, MA). To start, participants stood in the middle of the treadmill with one foot on each belt. A 12” tall partition was placed lengthwise between the belts to prohibit stepping on both belts simultaneously, but did not otherwise interfere with walking. Participants wore a safety harness suspended from the ceiling that did not provide body-weight support during walking. They were informed when the treadmill was about to start or stop, but were not told the structure of the paradigm (i.e. order of the testing blocks), the speed of the belts, or given instruction as to how to walk. Immediately prior to starting the treadmill, participants were allowed to hold on to a horizontal handrail in front of them but were instructed to release the rail and cross their arms as soon as possible. While walking, the participants watched a television placed directly in front of the treadmill to discourage them from looking at the treadmill belts. No feedback was provided on their performance. The treadmill was stopped briefly (<3 minutes) between each testing block (e.g. baseline, adapt 1, washout, etc.).

For all groups, participants walked at baseline with the belts tied alternating between slow and fast speeds in 2 minute increments (slow baseline 1, fast baseline 1, slow baseline 2). This was followed by a 10 minute adaptation block (adapt 1), a 25 minute washout block that alternated between the slow and fast speeds (slow washout 1 for 10 min, fast washout 1 for 5 min, slow washout 2 for 5 min, fast washout 2 for 5 min), and a second 10 minute adaptation block (adapt 2). Specific experimental protocols for each group are depicted in Figs 1 and 4.

### Data Collection

Kinematic data were collected at 100 Hz using Optotrak Certus motion capture hardware (Northern Digital, Waterloo, Ontario, Canada). Infared-emitting active markers were placed bilaterally over the tow (5th metatarsal head), ankle (lateral malleolus), knee (lateral femoral epicondyle), hip (greater trochanter), pelvis (iliac crest), and shoulder (acromion process). All participants remained on the treadmill throughout the duration of the testing sessions and wore comfortable walking shoes and form-fitting clothing to reduce marker movement artifact.

### Behavioral Data Analysis

For experiments 1 and 2, the outcome measure of interest was step length asymmetry (fast step length−slow step length/fast step length + slow step length). We calculated step length as the distance between the ankle markers along the anterior-posterior axis at heel strike of each leg. We analyzed SLA in adapt 1 and adapt 2 across four distinct time epochs: initial (mean of the first 5 strides), early change (mean of strides 6–30), late change (mean of strides 31–200), and the plateau (mean of the last 30 strides). In order to assess baseline behavior and the amount to which all groups were washed out following initial learning, we also assessed the plateau value of SLA during the fast baseline (fast baseline) and last washout (fast washout 2) blocks.

For both experiments, to confirm that no differences existed in baseline walking between the groups, we first performed separate one-way ANOVAs to compare baseline SLA values during walking at the slow speed (slow baseline 2). To assess the degree to which each group washed out the learning from adapt 1 before exposure to adapt 2, we performed mixed methods ANOVAs with a within-subject factor for time and a between subjects factor for group (α = 0.05) comparing the SLA at the plateau during fast baseline walking and last washout block (fast washout 2).

In Experiment 1, evaluation of the SLA in these time epochs allowed us to investigate savings by quantifying the differences between adapt 1 to adapt 2 (within and between groups) in the degree to which they were initially asymmetric (initial), the rate with which they learned to walk symmetrically (early and late change), and how symmetrically they walked by the end of learning (plateau). These comparisons were made using 2 × 3 (learning bout (adapt 1, adapt 2) x group (Small, Medium, Large)) mixed methods ANOVAs with the α level set to 0.05. When significant effects were observed, post hoc tests to compare adapt 1 to adapt 2 for each group were performed using Fisher’s LSD test. This post hoc test was chosen because multiple between group comparisons were not made, as such a post hoc correction for multiple comparisons was not warranted. Given that each group experienced difference belt speed configurations, no statistical analyses were performed to compare between group differences in SLA during these epochs within deadaptation (slow washout 1). We compared the level of symmetry at the end of adapt 1 using a one-way ANOVA to compare the SLA at plateau across the groups (α = 0.05). When significant effects were observed, post hoc tests were performed to compare among groups with a Bonferroni correction for multiple comparisons. Finally, we were also interested in the potential influence of symmetry achieved during adapt 1 on savings. To investigate this, we performed a one-way ANCOVA (α = 0.05) with the difference in late change from adapt 1 to adapt 2 as the dependent variable, group assignment as a fixed factor, and adapt 1 plateau as the covariate. When significant effects were observed, post hoc tests were performed to compare the adjusted group means with a Bonferroni correction for multiple comparisons.

Similarly, in Experiment 2 we calculated SLA in the same time epochs during adapt 2 for all groups. Adapt 2 data from the Small group in Experiment 1 were included in these analyses as reference data to allow for comparison of learning to that achieved with conventional savings paradigms. We compared these measures using one-way ANOVAs (α = 0.05). When significant effects were observed, post hoc tests were performed using a Dunnett’s test to allow for comparison of each group’s performance to the Small group reference data from Experiment 1. The Dunnett’s test was developed to make comparisons of multiple groups to a single reference or control. We employed it here in order to test the null hypothesis that no greater learning occurred relative to the reference group data from experiment 1. Since each group experienced different belt speed configurations during adapt 1 and deadaptation, there was no reasonable null hypothesis that all groups would perform similarly during these walking blocks. Therefore, no statistical analyses were performed on these data.

### Data Availability

The datasets analyzed during the current study are available from the corresponding author on reasonable request.
